# PD201 Rare Disease Product Approvals: The Changing Regulatory And Health Technology Assessment Landscape Between 2013 And 2022

**DOI:** 10.1017/S0266462324004239

**Published:** 2025-01-07

**Authors:** Tina Wang, Juan Lara, Bujar Magda, Belen Sola, Neil McAuslane

## Abstract

**Introduction:**

Globally, 7,000 rare diseases affect 300 million people, which poses challenges for developing treatments in these discrete patient populations. Developing medicines for rare diseases requires innovation, but despite regulatory incentives challenges persist for health technology assessment (HTA) and payers. Understanding the current regulatory and HTA decision-making landscape for orphan products is essential for all stakeholders.

**Methods:**

Data on new active substances (NAS) approvals (2013 to 2022) by the European Medicines Agency, the United States Food and Drug Administration (FDA), the Pharmaceuticals and Medical Devices Agency (PMDA), Swissmedic, and the Therapeutic Goods Administration were collected to analyze the timing, approval pathways, and global rollout trends for orphan and non-orphan products. Data were collected from HTA agencies in Australia, England, France, Germany, the Netherlands, Poland, Scotland, and Sweden to explore synchronization in decision timing and first HTA decision. Comparative analysis encompassed decision frameworks and funding mechanisms for orphan products among HTA agencies.

**Results:**

Orphan drug approvals increased in the past decade, with the FDA having the highest designation rate (55% for 2018 to 2022). Flexible pathways, mostly used by the FDA (92%) and the PMDA (100%), expedited orphan drug reviews. However, international submissions for orphan drugs experienced longer gaps than non-orphan drugs across the jurisdictions. Divergence in rollout timing to HTA and recommendation resulted from varied submission strategies and review processes. Only the Scottish Medicines Consortium had a dedicated orphan pathway. In England, the National Institute for Health and Care Excellence patient access and managed access agreements (84% of orphan recommendations) and cancer drug fund (45% of orphan recommendations) facilitated patient access.

**Conclusions:**

The study shows a decade long rise in global orphan drug approvals, underpinned by regulatory flexibility, particularly by the FDA and the PMDA. Identified divergences in decision frameworks among regulatory and HTA agencies, as well as HTA agencies themselves, call for increased stakeholder alignment. This necessitates synchronizing evidence generation during development and improving decision frameworks for streamlined review and reimbursement processes.

